# A pilot and comparative study between pathological and serological levels of immunoglobulin and complement among three kinds of primary glomerulonephritis

**DOI:** 10.1186/s12865-018-0254-z

**Published:** 2018-06-20

**Authors:** Jin Dong, Tianhao Peng, Jing Gao, Xingwang Jia, Guangtao Yan, Yong Wang

**Affiliations:** 1 Department of Clinical Biochemistry, Medical Laboratory Center, State Key Laboratory of Kidney Disease, Chinese PLA General Hospital, Beijing, 100853 China; 2 Testing Center of Health Management Institute, Chinese PLA General Hospital, Beijing, 100853 China; 3 Department of Nephrology, Chinese PLA Institute of Nephrology, State Key Laboratory of Kidney Diseases, National Clinical Research Center for Kidney Diseases, Medical Institution Conducting Clinical Trials, Chinese PLA General Hospital, Beijing, China

**Keywords:** Immunoglobulin A nephropathy, Membranous nephropathy, Minimal change disease, Immunoglobulin, Complement

## Abstract

**Background:**

Immunoglobulin A nephropathy (IgAN), membranous nephropathy (MN) and minimal-change disease (MCD) are three common types of glomerulonephritis in China. Pathological diagnosis based on renal biopsy is the criterion and the golden standard for diagnosing the sub-types of primary or secondary glomerulonephritis. Immunoglobulin and complements might be used in the differential diagnosis of glomerulonephritis without renal biopsies. However, the relationship between IF intensities of immune proteins and the corresponding serum levels remained unclear, and seldom studies combine histopathological examination results and blood tests together for a predictive purpose. This study was considered as a pilot study for integrating histopathological indicators into serum parameters for exploring the relationship of IF intensity and serum values of immunoglobulin and complement, and for screening and investigating effective indicators inIgAN, MN and MCD.

**Methods:**

Renal tissue immunofluorescence (IF) intensity grades and serum levels of immunoglobulin and complements (IgG, IgA, IgM, C3 and C4) were retrospectively analyzed in 236 cases with IgAN, MN or MCD. IF grades were grouped as negative (−), positive (+) or strong positive (++) with both high and low magnification of microscope. Other serum indicators such as urea nitrogen (BUN), creatinine (Crea) and estimated glomerular filtration rate (eGFR) were also evaluated among the groups.

**Results:**

There were difference in IgA, IgG and C3 IF intensity grades among IgAN, MN and MCD groups (*p* = 9.82E-43, 4.60E-39, 7.45E-15, respectively). Serum values of BUN, Crea, eGFR, IgG, IgA, IgM and C4 showed difference in three groups (BUN: *p* = 0.045, Crea: *p* = 3.45E-5, eGFR: *p* = 0.005, IgG: *p* = 1.68E-14, IgA: *p* = 9.14E-9, IgM: *p* = 0.014, C4: *p* = 0.026). eGFR had the trend to decrease with enhanced IgA IF positive grades (*p* = 8.99E-4); Crea had trends to decrease with both enhanced IgA and IgG IF intensity grades (*p* = 2.06E-6, 2.94E-5, respectively). In all subjects, serum IgA levels was inversely correlated with eGFR(*r* = − 0.216, *p* = 0.001) and correlated with Crea levels(*r* = 0.189, *p* = 0.004); serum IgG and Crea showed no correlation which were discordance with inverse correlation of IgG IF grade and Crea(*r* = 0.058,*p* = 0.379). IgG serum level was inverse correlated with its IF grades (*p* = 3.54E-5, *p* = 7.08E-6, respectively); C3 serum levels had significantly difference between Neg and positive (+) group (*p* = 0.0003). IgA serum level was positive correlated with its IF grades (Neg-(+): *p* = 0.0001; (+)-(++): *p* = 0.022; Neg-(++): *p* = 2.01E-10). After matching comparison among C3 groups, C3 Neg. group and C3 ++ group had difference (*p = 0.017). C4 had all negative IF expression in all pathological groups. In IgAN subjects, there were statistical differences of serum C3 levels between its pathological Neg and positive (+) group(*p* = 0.026), and serum IgA levels showed difference between IgA pathological positive(+) and (++)(*p* = 0.007). In MN subjects, sIgG levels showed difference between IgG pathological IF grade positive (+) and (++)(*p* = 0.044); serum C3 levels showed difference between C3 pathological IF grade Neg and positive(+)(*p* = 0.005); and serum IgA levels showed difference between Neg and positive(+)(*p* = 0.040). In IgAN, eGFR showed serum IgA levels had significant differences among groups (*p* = 0.007) and had increasing trend with enhanced its IF grades(P_trend_ = 0.016). There were also difference between IgG group Neg and positive (+) (*p* = 0.005, P_trend_ = 0.007) in IgAN. In MN, serum IgG levels had significant differences among IF groups (*p* = 0.034) and had decreasing trend with its enhanced IF grades (P_trend_ = 0.014). Serum C3 concentrations also were found distinctive among IF groups (*p* = 0.016) and had in inverse correlation with its enhanced IF grades (P_trend_ = 0.004).

**Discussion:**

Our research cross contrasts several immunoprotein IF intensities and relevant serum levels in three kinds of primary glomerular nephritis, and finally acquired helpful results for understanding the relationships between pathological presentation and serological presentation of immunoproteins in kidney diseases. Furthermore, this pilot study is offering a possible method for the analysis of combination of pathology and serology.

**Conclusion:**

Different pathological types of nephritis presented different expression patterns of immunoglobulin and complement, especially IgA and IgG, which suggested different pathogenesis involved in the development of IgAN and MN. Furthermore, either in tissue or in serum, increased IgA level was closely related with renal function in all of the patients.

**Electronic supplementary material:**

The online version of this article (10.1186/s12865-018-0254-z) contains supplementary material, which is available to authorized users.

## Background

Immunoglobulin A nephropathy (IgAN), membranous nephropathy (MN) and minimal-change disease (MCD) are three common types of glomerulonephritis in China [1, 2]. So far, pathological diagnosis based on invasive renal biopsy is the criterion and the golden standard for diagnosing the sub-types of primary or secondary glomerulonephritis, which is defined by the types of dominantly accumulated and infiltrated immunoglobulin in kidney tissue, such as IgAN is known as IgA or IgA-dominant immune complex deposition in glomerular mesangial through immunofluorescence. The formation and the deposition of immunoglobulin complexes play a key role in the pathogenesis of glomerulonephritis. An animal study on the progressive of glomerulonephropathy revealed that the formation of IgM deposition was earlier than of IgA and IgG deposition, and therefore IgA and IgG might be related with the progression of glomerular lesions [3]. Zhang et al. [4] found IgA/C3 changed with the progression of IgAN, whereas a study conducted by Yang et al. [5] showed decreased serum C3 level was not a leading factor in renal progression in IgAN. Mizerska-Wasiak M et al. [6] found that the IgA/C3 ratio was a useful marker for evaluating severity of lesions by Oxford classification in children with IgAN. All of those researches indicated immunoglobulin and complements might be used in the differential diagnosis of glomerulonephritis without renal biopsies. The evidence showed serum IgA value, serum IgA/C3 ratio and serum IgG value were related with IgAN or MN, so we supposed that blood markers might imitate histopathological changes and played a predictive role in diagnosis of the diseases. However, pathological parameters and sera parameters were always kept apart analysis in the most studies. The relationship between IF intensities of immune proteins and the corresponding serum levels remained unclear, and seldom studies combine histopathological examination results and blood tests together for a predictive purpose.

This study was considered as a pilot study for integrating histopathological indicators into serum parameters for exploring the relationship of IF intensity and serum values of immunoglobulin and complement, and for screening and investigating effective indicators for the diseases.

## Methods

### Subjects, inclusion and exclsion criteria and parameters

This study was a retrospective cohort study and was approved by the Medical Ethics Committee of the Chinese PLA general hospital. All patients had signed on the patient consents form for their agreements.

Patient’s inclusion criteria and exclusion criteria were described in Gao J’s study [7].

Data of two hundred and thirty-six patients who were diagnosed as IgAN, MN or MCD in Chinese PLA general hospital from Jan 1st, 2015 to Jan 1st, 2016 were retrospectively analyzed. The include parameters were as follows: IF intensity grades of immunoglobulin and complements (including IgG, IgM, IgA, C3, C4) with renal biopsies; serum levels of IgG, IgM, IgA, C3, C4, urea nitrogen (BUN) and creatinine (Crea); estimated glomerular filtration rate (eGFR) were also collected.

## Methods

**Fig. 1:**
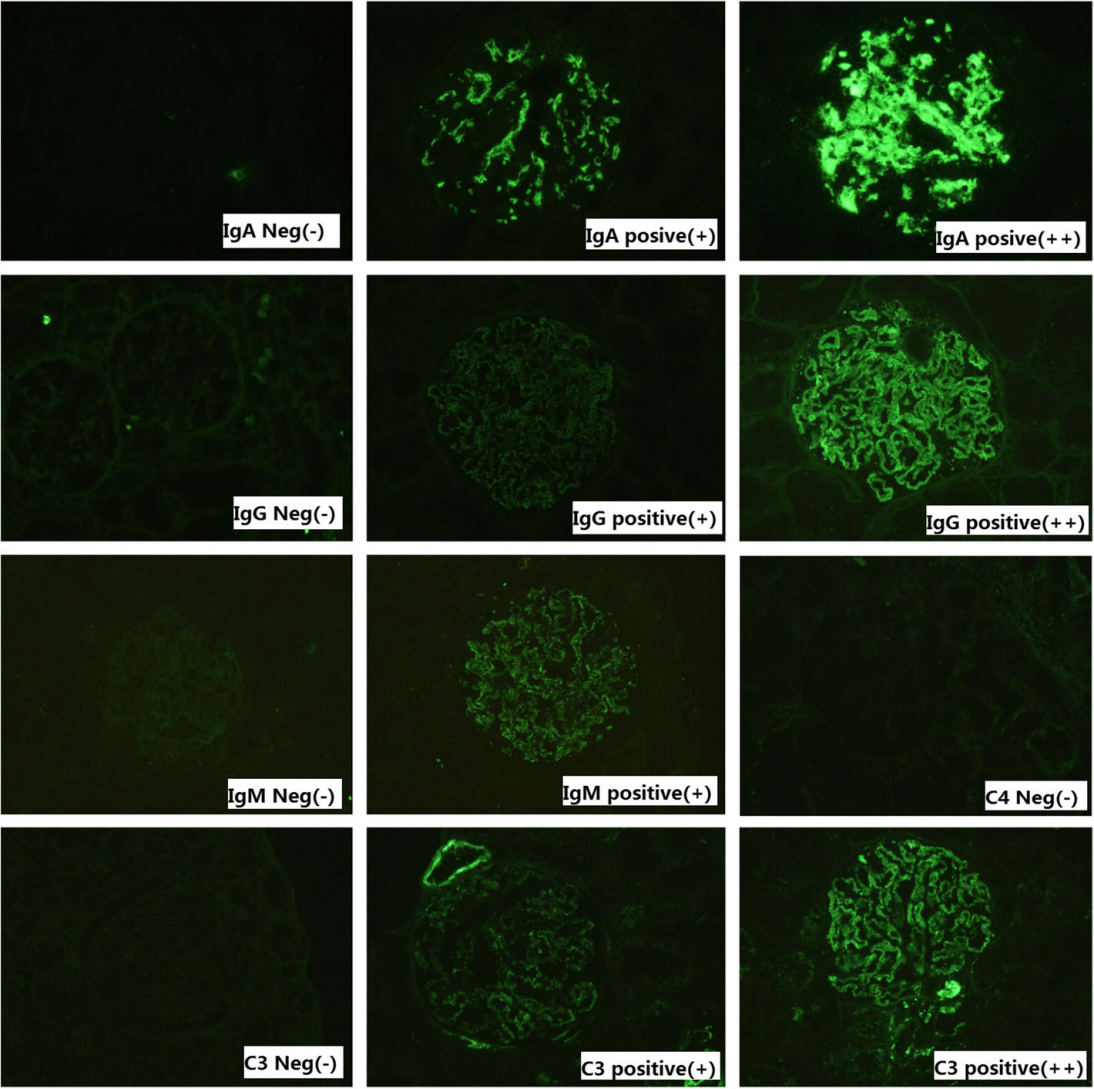
Immunofluorescence of negative, positive (+) and highly positive (++) of IgG, IgA, IgM, C3 and C4 (Magnification: X200). IF intensity grades of IgG, IgM,IgA, C3 and C4 were defined as follow: Neg: negative, +: positive, ++: strong positive

IF intensity grades of immunoglobulin and complement (IgG, IgM, IgA, C3, C4) were evaluated immunofluorescence of renal tissue, as a typical method of immunohistochemistry [8–10]. [As shown in Fig. 1, the classification of negative(Neg.), positive(+) and strong positive(++) was as follows: with immunofluorescence(high magnification:X200; low magnification: X10), negative(Neg.) is defined as: no fluorescence was observed in glomerular; positive(+) is defined as: fluorescence was observed by microscope with high magnification:X200, and fluorescence is faintly visible by microscope with low magnification: X10; positive(++) is defined as: fluorescence was clearly visible by microscope with high magnification:X200, and fluorescence is visible by microscope with low magnification: X10).]

Serum IgG, IgM, IgA, C3, C4 levels were tested on BNII analyzer (Siemens). Serum BUN and Crea were tested on Cobas analyzer (ROCHE). Estimated glomerular filtration rates (eGFR) (modified to Chinese population) were calculated [11].

### Groups divided

According to the diagnosis of renal biopsies, two hundred and thirty-six patients were divided into three groups: IgAN group (*n* = 88), MN group (*n* = 100), MCD group (*n* = 48). IF intensity grades were defined as negative (−), positive (+), and strong positive (++). Detailed characteristics of patients were shown in Table 1.

**Table 1:** General characteristics of patients in IgAN, MN and MCD groups

Parameters			IgAN	MN	MCD	Total	*P* value
Gender, n (female, %)			88(34.1)	100(41.0)	48(37.5)	236(100)	0.621
age, year(95% CI)			38.1(35.8–40.6)	48.3(45.3–51.2)	36.9(32.7–41.1)	–	6.34E-6
IF intensity grade	IgG	Neg	71(80.7%)	7(7.0%)	48(100.0%)	126(53.4%)	4.60E-39
		+	17(19.3%)	16(16.0%)	0(0.0%)	33(14.0%)	
		++	0(0.0%)	77(77.0%)	0(0.0%)	77(32.6%)	
	IgM	Neg	84(95.5%)	99(99.0%)	47(97.9%)	230(97.5%)	0.297
		+	4(4.5%)	1(1.0%)	1(2.1%)	6(2.5%)	
	IgA	Neg	0(0.0%)	91(91.0%)	48(100%)	139(58.9%)	9.82E-43
		+	32(36.4%)	5(5.0%)	0(0.0%)	37(15.7%)	
		++	56(63.6%)	4(4.0%)	0(0.0%)	60(25.4%)	
	C3	Neg	27(30.7%)	39(39.0%)	48(100.0%)	114(48.3%)	7.45E-15
		+	50(56.8%)	58(58.0%)	0(0.0%)	108(45.8%)	
		++	11(12.5%)	3(3.0%)	0(0.0%)	14(5.9)	
	C4	Neg	88(100.0%)	100(100.0%)	48(100.0%)	236(100.0%)	–
Serum values	Urea, mmol/L		6.27(5.63–6.92)	5.47(4.95–5.99)	6.25(5.13–7.38)	–	0.045
	Crea, μmol/L		111.6(100.1–123.2)	76.1(71.1–81.0)	81.7(75.6–87.7)		3.45E-5
	eGFR, ml/min per 1.73m^2^		83.2(76.2–90.3)	101.2(96.4–106.0)	103.4(96.0–110.8)		0.005
	sIgG, mg/dL		1015.4(952.6–1078.1)	619.0(565.3–672.6)	518.3(422.0–614.5)		1.68E-14
	sIgM, mg/dL		106.8(95.3–118.4)	129.1(110.4–147.9)	139.0(121.4–156.6)		0.014
	sIgA, mg/dL		308.0(285.9–330.1)	221.1(203.5–238.8)	211.7(185.4–237.9)		9.14E-9
	sC3, mg/dL		109.2(104.8–113.5)	116.0(111.0–120.9)	117.0(110.0–124.0)		0.124
	sC4, mg/dL		25.8(24.3–27.3)	29.9(27.8–32.0)	28.1(25.0–31.1)		0.026

Results were shown as mean(95% CI). *eGFR* was estimated by MDRD formula [1]. *IF* intensity grade of IgG, *IgM*, *IgA*, *C3* and *C4* was defined as following: Neg: negative, +: positive, ++: strong positive

### Statistics

Statistic analysis was performed on IBM SPSS Statistics software (version 19.0). Age and serum parameters were shown as means (95% confidence interval, 95% CI). Non-parametric tests (Mann-Whitney U test for comparison of two groups; Kruskal-Wallis H test for comparison of all Neg, positive (+) and strong positive (++) groups) and Chi-square test were used for the comparison among groups. P trend analysis was approximately evaluated by linear-by-linear association in Chi-square test. Correlation tests were used between serum immunoglobulin levels and renal function. *P* value under 0.05 was considered as a cut-off value as statistically significant difference.

## Results

### General characteristics of patients in IgAN, MN and MCD groups

As described in Table 1, there were differences in IgA, IgG and C3 IF intensity grades among IgAN, MN and MCD groups (*p* = 9.82E-43, 4.60E-39, 7.45E-15,respectively). Serum values of BUN, Crea, eGFR, IgG, IgA, IgM and C4 showed difference in three groups (BUN: *p* = 0.045, Crea: *p* = 3.45E-5, eGFR: *p* = 0.005, IgG: *p* = 1.68E-14, IgA: *p* = 9.14E-9, IgM: *p* = 0.014, C4: *p* = 0.026).

### Correlation of IF intensity grades and kidney function

Classification by IF intensity of immunoglobulin and complement as neg, positive (+) and strong positive (++) group, eGFR, BUN and Crea levels in each group were shown in Table 2. eGFR had the trend to decrease with enhanced IgA IF positive grades(*p* = 8.99E-4); Crea had trends to decrease with both enhanced IgA and IgG IF intensity grades (*p* = 2.06E-6, 2.94E-5,respectively). eGFR, BUN and Crea levels had no significantly changes among those groups.

**Table 2:** Comparison of kidney function among different IF grades of immunoglobulin and complement

Parameters		n	eGFR, ml/min per 1.73m^2^	P_trend_	Urea, mmol/L	P_trend_	Crea, μmol/L	P_trend_
IgG	Neg	126	89.7(84.1–95.4)	0.054	6.33(5.72–6.94)	0.167	100.7(92.0–109.5)	2.94E-5
	+	33	98.9(88.5–109.3)		5.42(4.32–6.51)		85.0(74.6–95.4)	
	++	77	101.7(96.5–107.0)		5.49(4.98–6.00)		76.0(70.7–81.3)	
IgM	Neg	230	95.2(91.4–99.0)	0.359	5.93(5.52–6.33)	0.326	90.0(84.6–95.4)	0.133
	+	6	83.6(49.2–117.9)		5.97(4.84–7.09)		107.7(67.2–148.2)	
IgA	Neg	139	102.1(98.1–106.1)	8.99E-4	5.75(5.21–6.27)	0.036	77.5(73.7–81.4)	2.06E-6
	+	37	75.8(64.6–87.0)		6.34(5.55–7.13)		117.7(101.5–133.8)	
	++	60	90.1(81.8–98.5)		6.09(5.25–6.93)		103.6(89.1–118.2)	
C3	Neg	114	99.4(94.2–104.7)	0.080	5.89(5.29–6.49)	0.269	85.3(78.7–92.0)	0.195
	+	108	90.3(84.4–96.2)		6.07(5.49–6.65)		96.1(87.2–104.9)	
	++	14	94.4(80.8–108.0)		5.18(4.14–6.22)		89.0(64.7–113.4)	
C4	Neg	236	94.9(91.2–98.7)	–	5.93(5.53–6.32)	–	90.5(85.1–95.8)	–

Results were shown as mean(95% CI). eGFR was estimated by MDRD formula [1]

*IF* intensity grades of IgG, I*gM,IgA*, *C3* and *C4* were defined as following: *Neg*: negative, +: positive, ++: strong positive

**Fig. 2:**
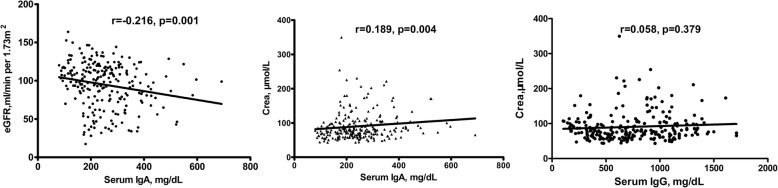
Correlation of serum IgA and IgG level with eGFR and Crea levels

Furthermore, we analyzed the correlation between eGFR, BUN, Crea levels and serum IgA, IgG levels. As shown in Fig. 2, in all subjects, serum IgA levels were inversely correlated with eGFR(*r* = − 0.216, *p* = 0.001) and correlated with Crea levels(*r* = 0.189, *p* = 0.004); serum IgG and Crea showed no correlation which were discordance with inverse correltion of IgG IF grade and Crea(*r* = 0.058,*p* = 0.379). An additional full parameters correlation analysis was shown as Additional file 1: Table S1.

In IgAN cases, not IgA but IgG was correlated to renal function indicators (serum IgG and eGFR: *r* = − 0.258, *p* = 0.015; serum IgA and Crea: *r* = 0.224, *p* = 0.036). In MN cases, only serum IgA was correlated with eGFR (*r* = − 0.234, *p* = 0.020) (Additional file 1: Table S1).

The cases included in this study were almost at the early stage of kidney disease (CKD1 or CKD2), so the relationship between IF intensity and relevant serum level was not investigated in the patients above CKD3 stage (eGFR< 60 ml/min per 1.73 m2). The observation in whole CKD stages is very important. However, there exists a contradiction in clinical that is end-stage renal disease is a contraindication for renal biopsy. It is not easy to observe the IF intensity changes of tissue immune proteins when patient’s renal function is very poor.

### Comparison of immunoprotein IF intensity grade and serum levels in total samples and in groups

**Fig. 3:**
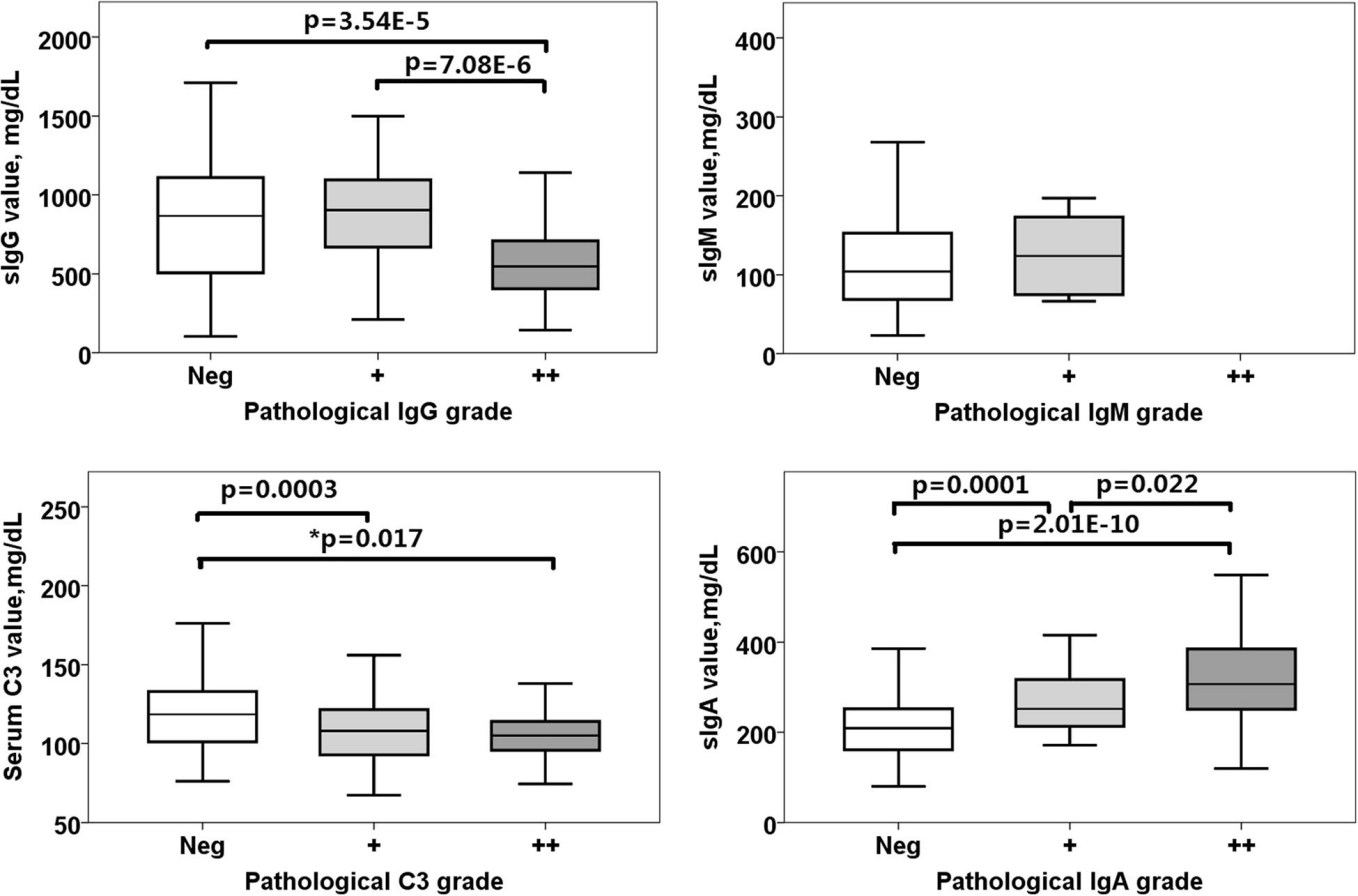
Comparison of immunoprotein IF intensities and relevant serum levels. Parameters with p or *p value under 0.05 were not listed. Pathological C3 group(++) (*n* = 14), for matching comparison with C3 group (Neg) and group (+), 20% samples were picked up randomly(*n* = 28, respectively).*p value were comparison of C3 Neg group(n = 28) and C3 (++)group. IF intensity grades of IgG, IgM,IgA, C3 and C4 were defined as follow: Neg: negative, +: positive, ++: strong positive

All immunoprotein IF grades and relevant serum levels were compared in all subjects. As shown in Fig. 3, in all subjects analysis, IgG serum level was inverse correlated with its IF intensity (i.e. serum IgG level decreased with enhanced IgG fluorescence intensity, *p* = 3.54E-5, *p* = 7.08E-6, respectively); C3 serum levels had significantly difference between Neg and positive (+) group (*p* = 0.0003). IgA serum level was positive correlated with its IF grades (i.e. serum IgA level increased with enhanced IgA IF grades, Neg-(+): *p* = 0.0001; (+)-(++): *p* = 0.022; Neg-(++): *p* = 2.01E-10). From Fig. 3, though C3 serum levels had trend to decrease in the pace with C3 IF intensity grade (Neg, +, ++), statistical analysis showed no significant difference (*p* = 0.067 in C3 Neg and C3 ++ group, *p* = 0.929 in C3 + and C3++ group). This contradict result may be caused by unbalanced sample numbers. As shown in Table 1, in ‘Total” column, 114 samples were in C3 Neg. group, 108 samples were in C3 + group and only 14 samples were in C3 ++ group. Statistical difference may be assumed with more C3++ samples. For matching comparison among C3 groups, we analyzed again. 20% samples from C3 Neg. group and C3 + group (*n* = 28, respectively) were randomly picked up, and then statistical significance appeared between C3 Neg. group and C3 ++ group (*p = 0.017). We have added the result into Fig. 3, labeled as *p. C4 had all negative IF expression in all pathological groups, thus C4 data was not listed in Fig. 3).

**Fig. 4:**
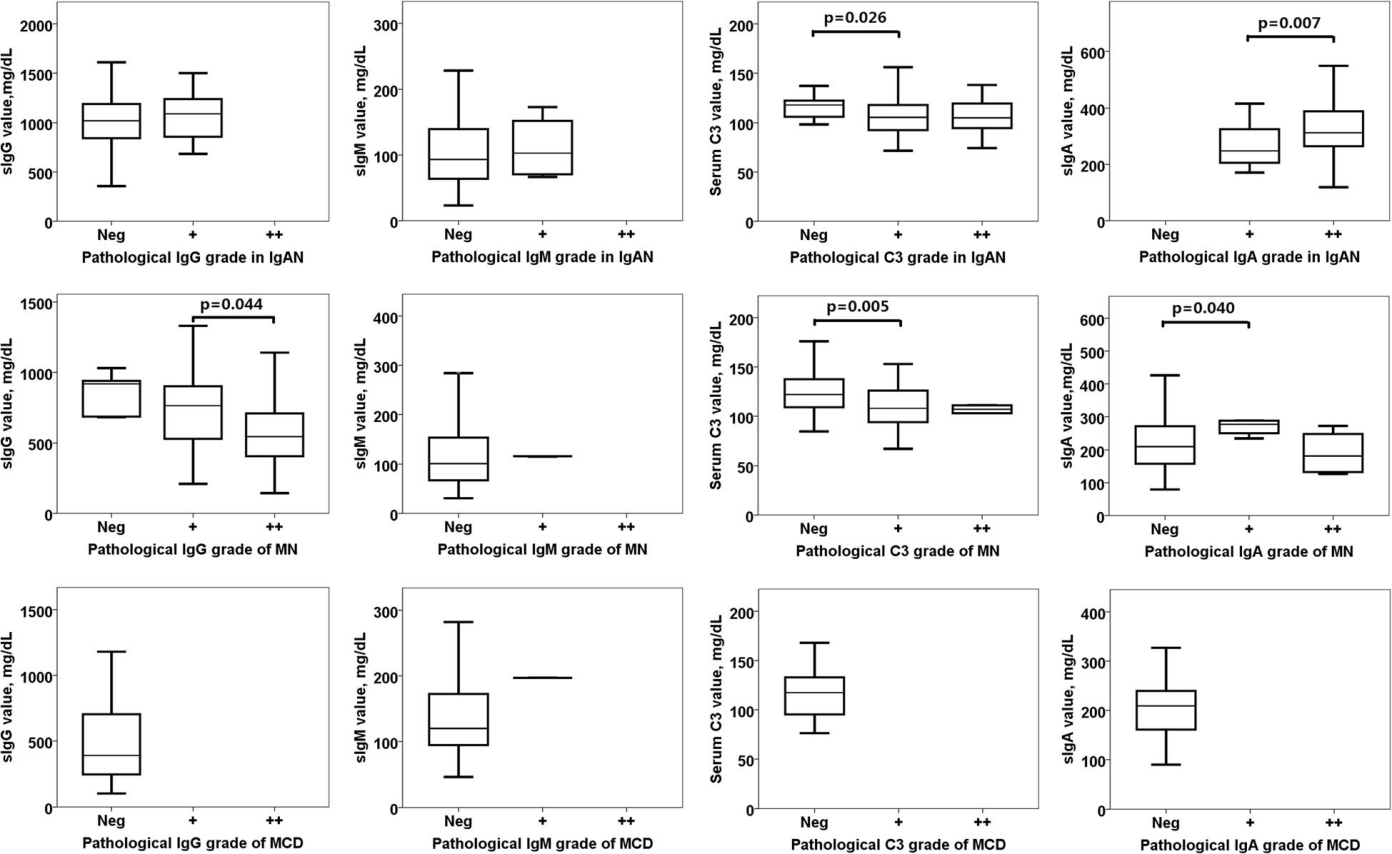
Comparison of IF intensity grades and serum levels of immunoglobulin and complement in group of IgAN, MN and MCD. Parameters with *p* value under 0.05 were not listed. IF intensity grades of IgG, IgM,IgA, C3 and C4 were defined as follow: Neg: negative, +: positive, ++: strong positive

We further explored all immunoprotein IF intensity grades and their serum values in IgAN, MN and MCD group (Fig. 4).

As shown in Fig. 4, the results showed that in IgAN subjects, there were statistical differences of serum C3 levels between its pathological Neg and positive(+) group(*p* = 0.026), and serum IgA levels showed difference between IgA pathological positive(+) and (++)(*p* = 0.007). In MN subjects, sIgG levels showed difference between IgG pathological If grade positive (+) and (++)(*p* = 0.044); serum C3 levels showed difference between C3 pathological IF grade Neg and positive(+)(*p* = 0.005); and serum IgA levels showed difference between Neg and positive(+)(*p* = 0.040). In MCD subjects, most of the IF intensities were pathologically reported negative, so the analysis could not be done.

Moreover, based on the analysis of pathological group comparisons of IgAN and MN, we further did trend correlation analysis for investigating the relationships of IgG, IgA and C3 IF intensity grades and their serum concentrations and renal function in IgAN and MN (Tables 3, 4 and 5). To confirm the uptrend or downtrend of those serum parameter values, we did an additional correlation analysis as Additional file 1: Table S2.

**Table 3:** Comparison of IF intensity grades and serum levels of IgG between IgAN and MN

	Parameters	IgG pathological grade			*P* value	P_trend_
		Neg	+	++		
IgAN	N(female)	71(26)	17(4)	–	0.234	–
	Age	39.7(36.9–42.4)	32.1(27.9–36.3)	–	0.014	0.015
	Un	6.52(5.74–7.29)	5.24(4.58–5.91)	–	0.129	0.120
	Cr	117.3(103.4–131.1)	88.1(76.3–99.9)	–	0.117	0.048
	eGFR	78.6(70.8–86.5)	102.6(89.1–116.1)	–	0.005	0.007
	sIgG	1013.2(942.7–1083.7)	1024.4(871.7–1177.1)	–	0.779	0.889
MN	N(female)	7(3)	16(8)	77(30)	0.712	–
	Age	51.0(42.2–59.8)	51.3(42.0–60.6)	47.4(43.9–50.8)	0.554	0.301
	Un	4.90(3.77–6.03)	5.68(3.22–8.14)	5.50(4.99–6.02)	0.555	0.703
	Cr	64.2(53.8--74.5)	82.8(62.7–103.0)	76.0(70.6–81.3)	0.383	0.601
	eGFR	108.8(96.2–121.5)	94.0(75.5–112.5)	101.6(96.4–106.9)	0.667	0.950
	sIgG	777.9(529.1–1026.6)	727.7(561.9–893.5)	582.9(525.0–640.7)	0.034	0.014

Pathological grades were *IF* intensity grades of IgG, *IgM*,*IgA*, *C3* and *C4* were defined as following: *Neg*: negative, +: positive, ++: strong positive. *P* value showed difference among Neg, positive (+) and (++)(analyzed by nonparametric tests-Kruskal-Wallis H test). P_trend_ showed serum immunoglobulin or compliment trends with their enhanced Pathological grade. P and P_trend_ values under 0.05 were considered as cut-off values as statistically significant differences

**Table 4:** Comparison of IF intensity grades and serum levels of IgA between IgAN and MN

	Parameters	IgA pathological grade			*P* value	P_trend_
		Neg	+	++		
IgAN	N(female)	–	32(9)	56(21)	0.257	–
	Age	–	41.8(36.9–46.6)	36.2(33.5–38.8)	0.039	0.028
	Un	–	6.46(5.57–7.34)	6.17(5.27–7.06)	0.288	0.669
	Cr	–	120.5(102.7–138.2)	106.6(91.3–121.9)	0.082	0.251
	eGFR	–	74.6(62.8–86.5)	88.2(79.5–96.9)	0.051	0.065
	sIgA	–	272.5(240.0–304.9)	328.3(299.5–357.1)	0.007	0.016
MN	N(female)	91(37)	5(3)	4(1)	0.556	–
	Age	48.7(45.4–51.9)	42.6(29.3–55.9)	45.3(34.1–56.5)	0.462	0.421
	Un	5.50(4.93–6.08)	5.61(3.27–7.96)	5.05(3.10–7.00)	0.815	0.822
	Cr	75.4(70.4–80.5)	99.9(46.4–153.5)	62.7(40.4–85.0)	0.212	0.908
	eGFR	101.2(96.3–106.2)	83.5(33.0–134.0)	117.5(99.9–135.0)	0.183	0.693
	sIgA	218.9(200.1–237.7)	285.0(218.0–352.0)	190.5(79.4–301.6)	0.092	0.847

Pathological grades were *IF* intensity grades of IgG, *IgM*,*IgA*, *C3* and *C4* were defined as following: *Neg*: negative, +: positive, ++: strong positive. *P* value showed difference among Neg, positive (+) and (++)(analyzed by nonparametric tests-Kruskal-Wallis H test). P_trend_ showed serum immunoglobulin or compliment trends with their enhanced Pathological grade. P and P_trend_ values under 0.05 were considered as cut-off values as statistically significant differences

**Table 5:** Comparison of IF intensity grades and serum levels of C3 between IgAN and MN

	Parameters	C3 pathological grade			*P* value	P_trend_
		Neg	+	++		
IgAN	N(female)	27(8)	50(19)	11(3)	0.668	–
	Age	37.1(32.6–41.6)	38.4(34.9–42.0)	39.9(35.7–44.2)	0.615	0.470
	Un	6.34(4.98–7.71)	6.40(5.54–7.26)	5.51(4.25–6.77)	0.510	0.567
	Cr	109.2(87.6–130.7)	116.8(100.8–132.7)	94.4(63.2–125.6)	0.346	0.703
	eGFR	86.9(73.5–100.4)	78.7(69.1–88.3)	94.9(77.6–112.2)	0.326	0.884
	sC3	115.6(108.6–122.7)	106.2(100.1–112.3)	106.9(93.0–120.8)	0.079	0.102
MN	N(female)	39(17)	58(22)	3(2)	0.562	–
	Age	49.5(45.1–53.9)	47.0(42.7–51.2)	57.3(42.2–72.5)	0.443	0.781
	Un	5.11(4.52–5.70)	5.80(4.99–6.62)	3.95(1.96–5.94)	0.252	0.514
	Cr	73.4(65.0–81.7)	78.3(71.5–85.0)	72.4(35.6–109.2)	0.323	0.525
	eGFR	103.2(94.9–111.4)	100.1(93.7–106.5)	83.6(0–179.5)	0.410	0.425
	sC3	125.0(116.9–133.2)	110.1(104.0–116.2)	107.0(56.2–157.8)	0.016	0.004

Pathological grades were *IF* intensity grades of IgG, *IgM*,*IgA*, *C3* and *C4* were defined as following: *Neg*: negative, +: positive, ++: strong positive. *P* value showed difference among Neg, positive (+) and (++)(analyzed by nonparametric tests-Kruskal-Wallis H test). P_trend_ showed serum immunoglobulin or compliment trends with their enhanced Pathological grade. P and P_trend_ values under 0.05 were considered as cut-off values as statistically significant differences

In IgAN, eGFR showed serum IgA levels had significant differences among groups(*p* = 0.007) and had increasing trend with enhanced its IF grades(P_trend_ = 0.016)(Table 4). There were also difference between IgG group Neg and positive (+) (*p* = 0.005, P_trend_ = 0.007) in IgAN(Table 3,IgAN).

In MN, serum IgG levels had significant differences among IF groups (*p* = 0.034) and was decreasing trend with its enhanced IF grades(P_trend_ = 0.014)(Table 3,MN). Serum C3 concentrations also were found distinctive among IF groups (*p* = 0.016) and had in inverse correlation with its enhanced IF grades(P_trend_ = 0.004)(Table 5,MN).

## Discussion

The morbidity of IgAN, MN and MCD has increased yearly, and IgAN is still dominant in primary glomerulonephritis [1]. Pathological results play decisive role in the diagnosis of nephropathy, by which kidney diseases are classified. Primary IgA nephropathy is characterized with IgA-dominant deposition in the glomerulus [12]; in MN, the pathological characteristics is IgG-dominant deposits [13] mostly with C3 deposits [14], and IgM or IgA has also been reported in MN [15, 16]; normally, less immonoprotein depositions are found in the patients with MCD [17]; few researches find that IgA, IgM, C3 or C1q immune complex could also exist in MCD [18–20].

Hence, the formation and the deposition of immunoprotein complexes are closely related with pathogenesis of primary glomerular nephritis. In our study, on analysis of all subjects, we found serum IgG and C3 concentrations trended to decrease with their enhanced IF grades, while serum IgA concentration elevated with the enhanced IgA IF intensity. Our analysis showed that in MN patients, serum IgG level declined with the enhanced IgG IF intensity. Interestingly, unlike changes of IgG, IgA in pathological level or serum level showed very different changes. Serum IgA value increased with the enhanced renal tissue IgA IF intensity. This may be related to the diversity mechanism of immune complex formation in IgAN and MN. Until now, the pathogenesis of IgAN remains unclear. Some scholars found that in the patients with IgAN, besides renal IgA complexes, there also was IgA complexes accumulating in the tissue of liver [12] or skin [21]. In the physiologic state, IgA can be synthesized in many tissues. IgA is composed of two subclasses as IgA1 and IgA2. Approximately 85 % IgA in serum is IgA1. IgA1 complexes are the main deposition in IgAN [22]. Therefore, the immune dysfunction in IgAN is involved not only in kidney but also in multiple systems and tissues. In our study, serum IgA (mainly IgA1) remaining high levels, that might be caused by the abnormal activation of immune system involved in multiple tissues, was correlated with the pathological IgA IF intensity grades. Multiple systems and tissues are involved in the changes. Some other observations also had conflict on initiating pathogenesis of IgAN [23, 24]. It is still unable to affirmed that which one is the initial tissue, kidney or others organs. Our analysis also indicated that C3 had synergistic effect in IgAN, which was correlated with serum IgA level. There were some reports found that IgA/C3 index, to some extent, was valuable in the diagnosis of IgAN [4, 6]. In MN patients, both serum IgG and C3 levels are reversely correlated with their respective IF intensity grades. The pathological characteristic of MN is the formation of immune complexes (mainly IgG and C3 complexes) deposit in glomerular capillary wall in situ [4]. Our analysis indicates that the IgG deposition might not source from circulating immune complexes: the formation of high levels of IgG-complexes had limited in nephridial tissue and less in other systems. In addition, the activation of complement system is also one of the factors of renal impairment. However, our results indicated that the pathogenesis of MN, unlike that of IgAN, might be an incipient and restrictive immune disease in kidney.

eGFR is a common indicator in clinic for estimation of renal function using gender, age and serum creatinine, which was modified by Ma, Y.C [11] for Chinese population. Our result showed that, there were decreased eGFR and elevated serum creatinine with positive IgA IF level, which was corresponding to trends of serum IgA level with eGFR and serum creatinine. The result indicated that the formation of IgA complexes aggravated dysfunction of kidney. Our result also showed that IgG IF intensity grade was correlated with serum creatinine, while irrelevant with eGFR. Since eGFR is calculated with several index (gender, age and serum creatinine), the factors of gender, age also affect calculation of eGFR; moreover, significant difference may be found with sample enlarged. In separate disease analysis, interestingly, we found that in IgAN patients (characteristic as positive IgA deposition), lower eGFR appeared in IgG negative patients than in IgG positive patients. As reported in some researches [8, 10, 25], in IgA nephropathy patients, IgG deposition showed at different degrees. In Nasri H′ reports, no significant association of IgG deposition with serum creatinine [10], and only C3 deposits had a significant correlation with serum creatinine [9]. In Wada Y’s study [8], proteinuria was greater in the both IgA-IgG positive group than the IgA positive group in IgAN patients and conclude that mesangial IgG deposition is associated with more severe clinical features in IgAN patients. The conflict of results of IgG and serum creatinine(and eFGR) may caused by following reasons: i. the subjects: As illustrated, the subjects included in this study were almost at the early stage of CKD1 or CKD2, and end-stage renal disease is a contraindication for renal biopsy. Besides, age might be another cause for age of IgG Neg group was 39.7(36.9–42.4) and age of positive (+) group was 32.1(27.9–36.3) in IgAN. ii. IgG positive grade: of all subjects in this study, we found no IgG strong positive(++) cases, while in Nasri H’s [10] and Wada Y’s [8] studies, there were cases involved with IgG IF strong positive grades. But from our present data, tissue IgG IF hardly showed strong positive in IgAN patients, which might caused by population diversities or IgG might played different role in pathogenesis and development of IgAN. The study implied that the positive rate of IgA and IgG complexes was relevant with renal function, in addition C3 might play roles in pathogenesis of primary glomerular nephritis, but was irrelevant with renal dysfunction.

Serum urea nitrogen and creatinine are most common indicators for estimation of renal function. In this study on IgG IF intensity grade, we found that serum creatinine decreased with increased IgG positive IF grades; while serum creatinine evaluated with increased IgA positive IF grades; same tendency was found in serological level. Serum IgG level was found uncorrelated with eGFR, nor serum urea nitrogen or creatinine. Zhang J, et al. [4] reported that serum IgA/C3 index had predictive effects on IgAN involvement. Our result showed that IgA level (from both serological and pathological levels) was closely related with renal function, and might affect prognosis of kidney. Otherwise, increased IgG level shows none of similar relationships in MN patients.

Medical diagnosis with artificial intelligence (AI) has been developed recent years. With BIG Data analysis (trend and risk analysis based on patients records and treatments), there are preliminary models set up for evaluation the risks, processes or diagnosis of disease [26, 27]. There are some diagnostic models that have now been set up and trained for differentiation of diseases based on ‘Big Data’, such as diagnosis of skin cancer by classification of skin lesions using deep convolutional neural networks [28], Big data analytics has been used in many fields in medicine, mostly in the health care of the illness or the health [29, 30]. As the golden standard for diagnosis of primary glomerular nephritis, histopatholgical examination is a invasive method including several steps of renal biopsy, sample collection, staining, and reading. However renal biopsy has limitations which are as follow: a. patients with the contraindication; b. case with medical disputes; c. adaptability and understanding of patients and their families. Our research cross contrasts several immunoprotein IF intensities and relevant serum levels in three kinds of primary glomerular nephritis, and finally acquired helpful results for understanding the relationships between pathological presentation and serological presentation of immunoproteins in kidney diseases. Furthermore, this pilot study is offering a possible method for the analysis of combination of pathology and serology.

## Conclusion

Our study showed that the changes of immunoprotein especially IgA and IgG, indicate the different pathogenesis of IgAN and MN, furthermore pathologically or serologically increased IgA level is closely related with renal function.

The limitation of this study is the sample numbers. Each group needs to be enlarged for more convince results. And some new renal disease markers, such as cystatin C and lipoprotein phospholipase A2, are not included.

## Additional file

Additional file 1: **Table S1.** Correlation between renal function and IgA or IgG. **Table S2.** Correlation between tissue IF and serum levels of immune proteins. Two supplemental Tables for explaining the revelation more clearly among IF, serum level and renal function. In **Table S1,** in all cases, eGFR and Crea were correlated to tissue IgA IF intensity grades, tissue IgG IF intensity grades and serum IgA level, respectively (serum IgA and eGFR: *r* = − 0.216, *p* = 0.001; serum IgA and Crea: *r* = 0.189, *p* = 0.004; IgA IF grades and eGFR: *r* = − 0.190, *p* = 0.003; IgA IF grades and Crea: *r* = 0.286, *p* = 0.000; IgG IF grades and eGFR: *r* = − 0.256, *p* = 0.000; IgG IF grades and Crea: *r* = 0.153, *p* = 0.018). As shown in **Table S2.** The results in the whole dataset, showed the highest correlation coefficient about 0.566 appeared in C3 and C4 serum levels, followed with 0.555 appeared in IgA IF intensity and serum IgG level, − 0.463 appeared in IgA and IgG IF grades, 0.452 appeared in IgA and IgG serum levels, and 0.449 appeared in IgA IF and relevant serum level. (DOCX 30 kb)

## Abbreviations

BUN: Urea nitrogen
C3: Complement C3
C4: Complement C4
Crea: Creatinine
eGFR: estimated Glomerular Filtration Rate
IF: Immunofluorescence
IgA: Immunoglobulin A
IgAN: Immunoglobulin A nephropathy
IgG: Immunoglobulin G
IgM: Immunoglobulin M
MCD: Minimal-change disease
MN: Membranous nephropathy
