# Supplementary material for: A pilot and comparative study between pathological and serological levels of immunoglobulin and complement among three kinds of primary glomerulonephritis
Source: BMC Immunol. 2018 Jun 20;19:18. doi: 10.1186/s12865-018-0254-z (PMC6011399; doi:10.1186/s12865-018-0254-z)
Supplement: Supplementary file 1 — Table S1. Correlation between renal function and IgA or IgG. Table S2. Correlation between tissue IF and serum levels of immune proteins. Two supplemental Tables for explaining the revelation more clearly among IF, serum level and renal function. In Table S1, in all cases, eGFR and Crea were correlated to tissue IgA IF intensity grades, tissue IgG IF intensity grades and serum IgA level, respectively (serum IgA and eGFR: r = − 0.216, p = 0.001; serum IgA and Crea: r = 0.189, p = 0.004; IgA IF grades and eGFR: r = − 0.190, p = 0.003; IgA IF grades and Crea: r = 0.286, p = 0.000; IgG IF grades and eGFR: r = − 0.256, p = 0.000; IgG IF grades and Crea: r = 0.153, p = 0.018). As shown in Table S2. The results in the whole dataset, showed the highest correlation coefficient about 0.566 appeared in C3 and C4 serum levels, followed with 0.555 appeared in IgA IF intensity and serum IgG level, − 0.463 appeared in IgA and IgG IF grades, 0.452 appeared in IgA and IgG serum levels, and 0.449 appeared in IgA IF and relevant serum level. (DOCX 30 kb) [file 12865_2018_254_MOESM1_ESM.docx]

Table S1 Correlation between renal function and IgA or IgG

|  |  | **In all cases** | | **In IgAN** | | **In MN** | |
| --- | --- | --- | --- | --- | --- | --- | --- |
|  |  | **Cr** | **eGFR** | **Cr** | **eGFR** | **Cr** | **eGFR** |
| **PIgA** | Correlation Coefficient | .286^**^ | -.190^**^ | -.186 | .209 | .026 | .062 |
|  | Sig. (2-tailed) | .000 | .003 | .082 | .050 | .795 | .539 |
|  | N | 236 | 236 | 88 | 88 | 100 | 100 |
| **IgA** | Correlation Coefficient | .189^**^ | -.216^**^ | -.091 | .043 | .145 | -.234^*^ |
|  | Sig. (2-tailed) | .004 | .001 | .399 | .690 | .155 | .020 |
|  | N | 234 | 234 | 88 | 88 | 98 | 98 |
| **PIgG** | Correlation Coefficient | -.256^**^ | .153^*^ | -.168 | .299^**^ | .084 | -.010 |
|  | Sig. (2-tailed) | .000 | .018 | .117 | .005 | .405 | .918 |
|  | N | 236 | 236 | 88 | 88 | 100 | 100 |
| **IgG** | Correlation Coefficient | .058 | -.095 | -.258^*^ | .224^*^ | -.073 | -.047 |
|  | Sig. (2-tailed) | .379 | .146 | .015 | .036 | .472 | .648 |
|  | N | 234 | 234 | 88 | 88 | 98 | 98 |

Table S2 Correlation between tissue IF and serum levels of immune proteins

|  |  |  | **sIgA** | **PIgA** | **PIgG** | **sIgG** | **sPC3** | **sC3** | **PC4** | **C4** |
| --- | --- | --- | --- | --- | --- | --- | --- | --- | --- | --- |
| **In all cases** | **sIgA** | Correlation Coefficient | 1.000 | .449^**^ | -.182^**^ | .452^**^ | .297^**^ | .068 | . | .043 |
|  |  | Sig. (2-tailed) | . | .000 | .005 | .000 | .000 | .298 | . | .515 |
|  |  | N | 234 | 234 | 234 | 234 | 234 | 234 | 234 | 234 |
|  | **PIgA** | Correlation Coefficient | .449^**^ | 1.000 | -.463^**^ | .555^**^ | .269^**^ | -.101 | . | -.168^**^ |
|  |  | Sig. (2-tailed) | .000 | . | .000 | .000 | .000 | .125 | . | .010 |
|  |  | N | 234 | 236 | 236 | 234 | 236 | 234 | 236 | 234 |
|  | **PIgG** | Correlation Coefficient | -.182^**^ | -.463^**^ | 1.000 | -.251^**^ | .244^**^ | .060 | . | .136^*^ |
|  |  | Sig. (2-tailed) | .005 | .000 | . | .000 | .000 | .364 | . | .037 |
|  |  | N | 234 | 236 | 236 | 234 | 236 | 234 | 236 | 234 |
|  | **sIgG** | Correlation Coefficient | .452^**^ | .555^**^ | -.251^**^ | 1.000 | .150^*^ | -.009 | . | -.164^*^ |
|  |  | Sig. (2-tailed) | .000 | .000 | .000 | . | .022 | .895 | . | .012 |
|  |  | N | 234 | 234 | 234 | 234 | 234 | 234 | 234 | 234 |
|  | **PC3** | Correlation Coefficient | .297^**^ | .269^**^ | .244^**^ | .150^*^ | 1.000 | -.241^**^ | . | -.123 |
|  |  | Sig. (2-tailed) | .000 | .000 | .000 | .022 | . | .000 | . | .061 |
|  |  | N | 234 | 236 | 236 | 234 | 236 | 234 | 236 | 234 |
|  | **C3** | Correlation Coefficient | .068 | -.101 | .060 | -.009 | -.241^**^ | 1.000 | . | .566^**^ |
|  |  | Sig. (2-tailed) | .298 | .125 | .364 | .895 | .000 | . | . | .000 |
|  |  | N | 234 | 234 | 234 | 234 | 234 | 234 | 234 | 234 |
|  | **PC4** | Correlation Coefficient | . | . | . | . | . | . | . | . |
|  |  | Sig. (2-tailed) | . | . | . | . | . | . | . | . |
|  |  | N | 234 | 236 | 236 | 234 | 236 | 234 | 236 | 234 |
|  | **C4** | Correlation Coefficient | .043 | -.168^**^ | .136^*^ | -.164^*^ | -.123 | .566^**^ | . | 1.000 |
|  |  | Sig. (2-tailed) | .515 | .010 | .037 | .012 | .061 | .000 | . | . |
|  |  | N | 234 | 234 | 234 | 234 | 234 | 234 | 234 | 234 |
| **In IgAN** | **sIgA** | Correlation Coefficient | 1.000 | .287^**^ | -.118 | .343^**^ | .284^**^ | .181 | . | -.032 |
|  |  | Sig. (2-tailed) | . | .007 | .272 | .001 | .007 | .092 | . | .768 |
|  |  | N | 88 | 88 | 88 | 88 | 88 | 88 | 88 | 88 |
|  | **PIgA** | Correlation Coefficient | .287^**^ | 1.000 | .190 | -.002 | .120 | .143 | . | -.157 |
|  |  | Sig. (2-tailed) | .007 | . | .076 | .983 | .265 | .184 | . | .145 |
|  |  | N | 88 | 88 | 88 | 88 | 88 | 88 | 88 | 88 |
|  | **PIgG** | Correlation Coefficient | -.118 | .190 | 1.000 | .030 | .104 | .120 | . | -.012 |
|  |  | Sig. (2-tailed) | .272 | .076 | . | .781 | .334 | .265 | . | .912 |
|  |  | N | 88 | 88 | 88 | 88 | 88 | 88 | 88 | 88 |
|  | **sIgG** | Correlation Coefficient | .343^**^ | -.002 | .030 | 1.000 | -.063 | .167 | . | -.032 |
|  |  | Sig. (2-tailed) | .001 | .983 | .781 | . | .562 | .121 | . | .770 |
|  |  | N | 88 | 88 | 88 | 88 | 88 | 88 | 88 | 88 |
|  | **PC3** | Correlation Coefficient | .284^**^ | .120 | .104 | -.063 | 1.000 | -.210 | . | -.165 |
|  |  | Sig. (2-tailed) | .007 | .265 | .334 | .562 | . | .050 | . | .126 |
|  |  | N | 88 | 88 | 88 | 88 | 88 | 88 | 88 | 88 |
|  | **sC3** | Correlation Coefficient | .181 | .143 | .120 | .167 | -.210 | 1.000 | . | .435^**^ |
|  |  | Sig. (2-tailed) | .092 | .184 | .265 | .121 | .050 | . | . | .000 |
|  |  | N | 88 | 88 | 88 | 88 | 88 | 88 | 88 | 88 |
|  | **PC4** | Correlation Coefficient | . | . | . | . | . | . | . | . |
|  |  | Sig. (2-tailed) | . | . | . | . | . | . | . | . |
|  |  | N | 88 | 88 | 88 | 88 | 88 | 88 | 88 | 88 |
|  | **sC4** | Correlation Coefficient | -.032 | -.157 | -.012 | -.032 | -.165 | .435^**^ | . | 1.000 |
|  |  | Sig. (2-tailed) | .768 | .145 | .912 | .770 | .126 | .000 | . | . |
|  |  | N | 88 | 88 | 88 | 88 | 88 | 88 | 88 | 88 |
| **In MN** | **sIgA** | Correlation Coefficient | 1.000 | .104 | .040 | .341^**^ | .133 | .099 | . | .148 |
|  |  | Sig. (2-tailed) | . | .306 | .696 | .001 | .192 | .332 | . | .147 |
|  |  | N | 98 | 98 | 98 | 98 | 98 | 98 | 98 | 98 |
|  | **PIgA** | Correlation Coefficient | .104 | 1.000 | -.304^**^ | .135 | -.189 | .012 | . | -.056 |
|  |  | Sig. (2-tailed) | .306 | . | .002 | .186 | .060 | .903 | . | .583 |
|  |  | N | 98 | 100 | 100 | 98 | 100 | 98 | 100 | 98 |
|  | **PIgG** | Correlation Coefficient | .040 | -.304^**^ | 1.000 | -.264^**^ | .294^**^ | -.051 | . | .032 |
|  |  | Sig. (2-tailed) | .696 | .002 | . | .009 | .003 | .617 | . | .751 |
|  |  | N | 98 | 100 | 100 | 98 | 100 | 98 | 100 | 98 |
|  | **sIgG** | Correlation Coefficient | .341^**^ | .135 | -.264^**^ | 1.000 | -.203^*^ | .132 | . | -.002 |
|  |  | Sig. (2-tailed) | .001 | .186 | .009 | . | .044 | .195 | . | .981 |
|  |  | N | 98 | 98 | 98 | 98 | 98 | 98 | 98 | 98 |
|  | **PC3** | Correlation Coefficient | .133 | -.189 | .294^**^ | -.203^*^ | 1.000 | -.291^**^ | . | -.137 |
|  |  | Sig. (2-tailed) | .192 | .060 | .003 | .044 | . | .004 | . | .179 |
|  |  | N | 98 | 100 | 100 | 98 | 100 | 98 | 100 | 98 |
|  | **sC3** | Correlation Coefficient | .099 | .012 | -.051 | .132 | -.291^**^ | 1.000 | . | .628^**^ |
|  |  | Sig. (2-tailed) | .332 | .903 | .617 | .195 | .004 | . | . | .000 |
|  |  | N | 98 | 98 | 98 | 98 | 98 | 98 | 98 | 98 |
|  | **PC4** | Correlation Coefficient | . | . | . | . | . | . | . | . |
|  |  | Sig. (2-tailed) | . | . | . | . | . | . | . | . |
|  |  | N | 98 | 100 | 100 | 98 | 100 | 98 | 100 | 98 |
|  | **sC4** | Correlation Coefficient | .148 | -.056 | .032 | -.002 | -.137 | .628^**^ | . | 1.000 |
|  |  | Sig. (2-tailed) | .147 | .583 | .751 | .981 | .179 | .000 | . | . |
|  |  | N | 98 | 98 | 98 | 98 | 98 | 98 | 98 | 98 |

Note: PIgs means pathological IF grades of IgG, IgM ,IgA, C3 and C4.
